# On the Gas-Phase Interactions of Alkyl and Phenyl Formates with Water: Ion–Molecule Reactions with Proton-Bound Water Clusters

**DOI:** 10.3390/molecules28114431

**Published:** 2023-05-30

**Authors:** Malick Diedhiou, Paul M. Mayer

**Affiliations:** Department of Chemistry and Biomolecular Sciences, University of Ottawa, Ottawa, ON K1N 6N5, Canada; mdiedhio@uottawa.ca

**Keywords:** formates, proton-bound water clusters, ion-molecular reactions, mass spectrometry, collision-induced dissociation, RRKM theory, computational chemistry

## Abstract

Ion–molecule reactions between the neutral ethyl- (EF), isopropyl- (IF), t-butyl- (TF) and phenyl formate (PF) and proton-bound water clusters W_2_H^+^ and W_3_H^+^ (W = H_2_O) showed that the major reaction product is water loss from the initial encounter complex, followed ultimately by the formation of the protonated formate. Collision-induced dissociation breakdown curves of the formate–water complexes were obtained as a function of collision energy and modeled to extract relative activation energies for the observed channels. Density functional theory calculations (B3LYP/6-311+G(d,p)) of the water loss reactions were consistent with reactions having no reverse energy barrier in each case. Overall, the results indicate that the interaction of formates with atmospheric water can form stable encounter complexes that will dissociate by sequential water loss to form protonated formates.

## 1. Introduction

Small water clusters are believed to play a crucial role in the catalysis of atmospheric chemical reactions [1]. Studies have shown that clusters of different sizes (W_n_, where n represents the number of water molecules in the cluster) can interconvert through fast equilibration [2,3]. The ability of water molecules to act both as hydrogen bond donors and acceptors allows water clusters in the atmosphere to catalyze a plethora of chemical reactions by stabilizing intermediates and reducing transition state free energies. These reactive species are responsible for downstream atmospheric reactions such as the oxidation of reactive organic carbon (ROC) and volatile organic carbon (VOC) [4,5].

ROCs and VOCs are chemicals released from industrial processes or generated from the burning of fossil fuels and are known contributors to atmospheric pollution. However, the specific contributions of various ROC and VOC species, and the factors affecting their concentrations, are still not yet fully understood [6,7]. Generally, VOCs are known to be important carcinogens and important gaseous precursors in the photochemical generation of ozone in the troposphere. The importance of VOCs in the atmosphere and their ability to affect climate change, their health effects, and oxidation capacity have already been discussed and studied extensively [8]. Cluster ions containing water and small ROCs and VOCs have been an area of interest in atmospheric chemistry for over 30 years [9,10].

Formate compounds are known to be one of the most abundant oxygen-rich species in the atmosphere. Known sources of these species include industrial solvents, biogenic sources, secondary oxidation, biomass burning, and vehicle exhaust (incomplete combustion and fuel additives) [11,12,13,14]. After being emitted, formate-derived esters can undergo a range of chemical reactions in the atmosphere, including a series of oxidation reactions in the troposphere initiated by oxidants, notably the hydroxyl radical [15,16,17,18,19]. Formate concentrations in non-urban precipitation were reported to be 10 to 30 µM [20,21,22], while Khare et al. demonstrated they are also present in cloud water, fog, and dew [14], leading to the main sinks for formates being wet and dry deposition. This indicates that the interaction of atmospheric formates with water is a significant aspect of their gas-phase chemistry. The reaction between a formate and a proton source such as water can lead to proton transfer, which results in the formation of the protonated formate [23,24].

The nature of the water cluster, specifically its hydrogen bonding, allows the prediction that the reaction between a water cluster and a ROC species should involve three main steps: the formation of the pre-reactive complex (RC), formation of the product complex (PC), and lastly dissociation to ultimately form a protonated ROC [23].

**Figure 1:**
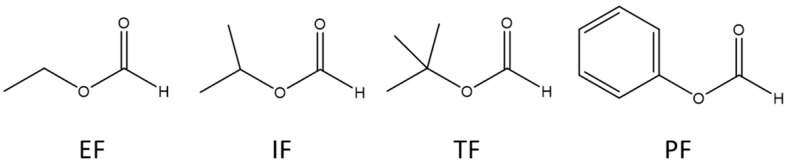
Structures of the four formates explored in this study.

We previously explored the interaction of methyl formate with protonated water, methanol, and ethanol clusters using reactive tandem mass spectrometry [23]. The primary reaction of the initially formed encounter complex was to lose successive solvent molecules to form protonated methyl formate. However, other reactions were also observed that were a function of the methyl group of methyl formate and the proton affinity (PA) difference between MF and the solvent molecule. Thus, in this study, we aimed to explore the interaction of a series of atmospherically relevant formates with varying substituents: ethyl formate (EF), isopropyl formate (IF), tertbutyl formate (TF), and phenyl formate (PF), shown in Figure 1, with proton-bound water clusters (H_2_O)_2_H^+^ and (H_2_O)_3_H^+^ using reactive tandem mass spectrometry. The role of both the side chain and overall PA on the observed reactivity could be assessed. The unimolecular dissociation pathways for some of the generated reactive complexes were explored experimentally with collision-induced dissociation (CID) mass spectrometry and computationally with density functional theory.

## 2. Results and Discussion

### 2.1. Water Cluster Ion/Formate Reactions

**Figure 2:**
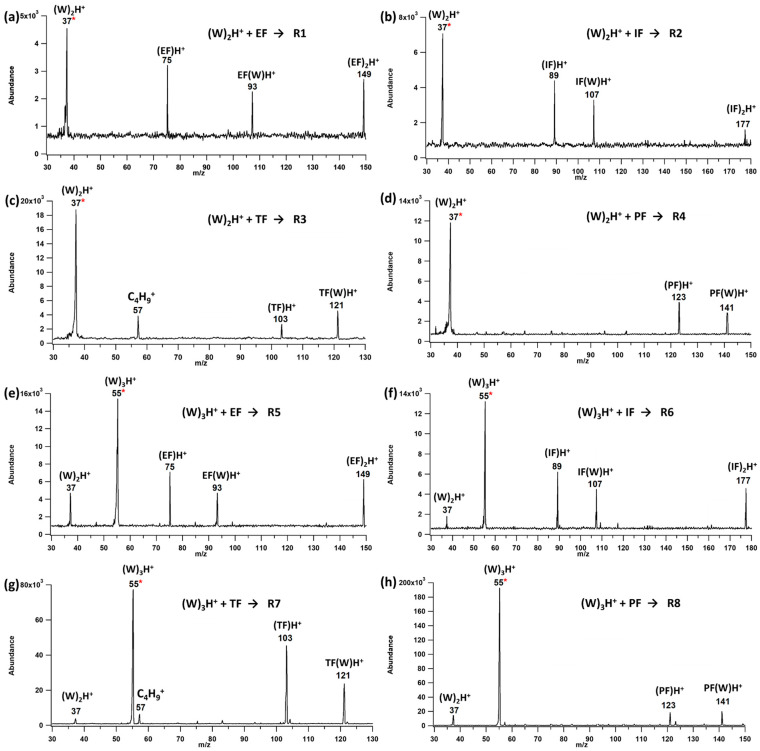
Representative mass spectra of protonated water dimer ion (*m*/*z* 37, highlighted with the *) and protonated water trimer ion (*m*/*z* 55, highlighted with the *) reacting with neutral (**a**,**e**) EF (74 Da, **R1**, **R5**), (**b**,**f**) IF (88 Da, **R2**, **R6**), (**c**,**g**) TF (102 Da, **R3**, **R7**), (**d**,**h**), and PF (122 Da, **R4**, **R8**). In each case the collision energy was set to 1 eV.

Figure 2 displays representative mass spectra from ion–molecule reactions **R1**–**R8** between proton-bound water clusters (dimers and trimers) and neutral ethyl, isopropyl, t-butyl, and phenyl formate. Water = W, ethyl formate = EF, isopropyl formate = IF, t-butyl formate = TF, and phenyl formate = PF.

The mass spectra from the reaction between protonated water dimer ions and ethyl formate (**R1**) revealed multiple peaks, including *m*/*z* 93, which is the proton-bound water–ethyl formate complex; *m*/*z* 75, which would nominally be protonated EF; and *m*/*z* 149 representing the proton-bound EF dimer, (EF)_2_H^+^. The observation of protonated formate and the proton-bound formate–water complex was common to all reactions. For TF, *m*/*z* 57 was also observed, which is likely the t-butyl cation, a known fragment of protonated TF [24]. Interestingly, another abundant fragment of TFH^+^, *m*/*z* 71 (TFH^+^-CH_3_OH), was not observed. This is a higher energy process and the present results indicate that the loss of water leaves the resulting TFH^+^ with a low enough internal energy distribution to preclude the participation of this channel. Similarly, Figure 2 shows representative mass spectra resulting from the ion–molecule reactions of each formate with the proton-bound water trimer ions (*m*/*z* 55). The same reaction products are observed from the trimer as were observed from the reactions with the dimer. In each case the dominant products are the protonated formate and the proton-bound formate–water complex. For EF and IF, the proton-bound formate dimer is also observed.

**Figure 3:**
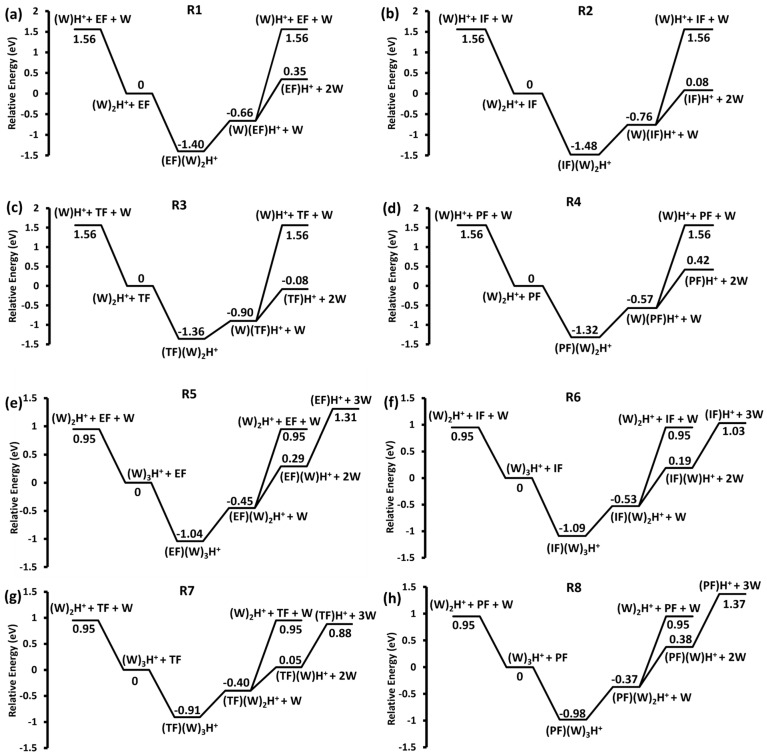
Relative energy (B3LYP/6-311+G(d,p)) of the main product ions from the interaction of the protonated water dimer ion (*m*/*z* 37) with neutral (**a**) EF (**R1**), (**b**) IF (**R2**), (**c**) TF (**R3**), and (**d**) PF (**R4**) and protonated trimer ion (*m*/*z* 55) with neutral (**e**) EF (**R5**), (**f**) IF (**R6**), (**g**) TF (**R7**), and (**h**) PF (**R8**).

The relative energy of the reaction products for the formation and dissociation of the encounter complexes was calculated at the B3LYP/6-311+G(d,p) level of theory and is shown in Figure 3. The protonated water dimer and trimer ions were calculated to have the structure predicted by Duong and co-workers (Figure S1) [25]. The initially generated encounter complexes lie between 0.91 and 1.4 eV below the reactants. In each case, the subsequent loss of water from the encounter complex remains exothermic with respect to the initial reactants. For reactions **R5**–**R8**, the loss of a second water molecule is only slightly endothermic in each case. According to the relative energies, it is most likely that any observed WH^+^ results from the dissociation of the original (W)_2_H^+^ complex, since it would not compete favorably, from EF(W)H^+^, IF(W)H^+^, TF(W)H^+^, or PF(W)H^+^, which form the protonated formate in each case. Likewise, in **R5**–**R8** the (W)_2_H^+^ ion results from the dissociation of (W)_3_H^+^.

**Figure 4:**
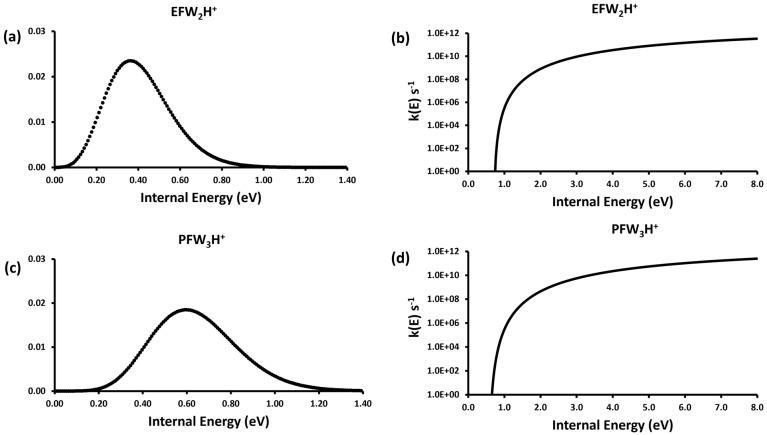
Vibrational internal energy distributions at 300 K for the encounter complexes in (**a**) **R1** and (**c**) **R8** and the RRKM *k*(*E*) vs. E curves for the dissociation of the corresponding ion–molecule encounter complexes (**b**,**d**).

The encounter complexes were not detected in the experiment because their formation is exothermic with respect to the initial reactants and the resulting loss of W is also exothermic. For example, the predicted dissociation energies for the EF(W)_2_H^+^ and PF(W)_3_H^+^ encounter complexes are 0.74 eV and 0.61 eV, respectively. The encounter complexes in a thermal system at 300 K exhibit an internal energy distribution that extends to 1.2 eV (Figure 4a,c), indicating that they would be stable species under atmospheric pressure. In the current experiment, however, the initial encounter complex is not seen because there is not enough time or pressure to collisionally stabilize the complex with respect to water loss. For both ions (and indeed for all the ions studied here) the microcanonical rate constant for water loss from the initially formed reactive complex at the internal energy with which they are formed is greater than 10^8^ s^−1^ (Figure 4b,d). As described above, at higher pressures closer to 1 atm collisional stabilization would be fast enough to form a stable population of the encounter complexes. The overall primary sequence of reactions observed upon the reaction of a water cluster with a gas-phase formate molecule is to form an encounter complex that can readily lose water. In the atmosphere the reaction would likely stop at this this point as the re-equilibrated product ion would have insufficient internal energy to dissociate further. In the mass spectrometer, the dissociation can continue to form the protonated formate.

### 2.2. Unimolecular Reactions of Proton-Bound Water–Formate Clusters

**Figure 5:**
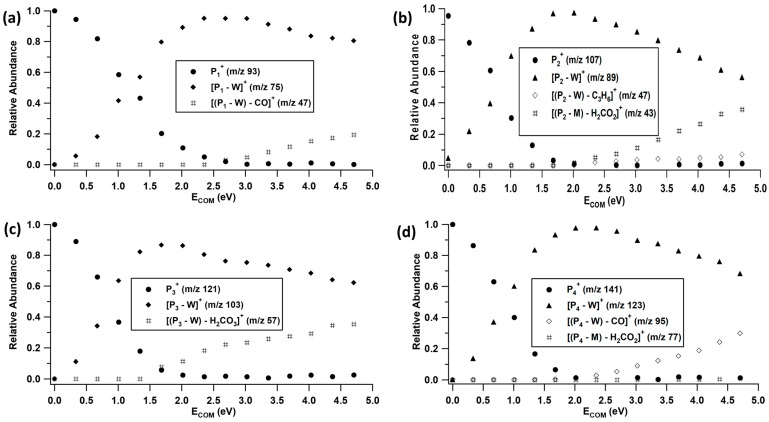
CID breakdown curves of (**a**) P_1_^+^ = EF(W)H^+^, (**b**) P_2_^+^ = IF(W)H^+^, (**c**) P_3_^+^ = TF(W)H^+^, and (**d**) P_4_^+^ = PF(W)H^+^.

The proton-bound complexes between EF, IF, TF, and PF and a water molecule are the most prevalent principal reaction products observed in the ion–molecule reactions. In the electrospray source, we were able to independently produce EF(W)H^+^, IF(W)H^+^, TF(W)H^+^, and PF(W)H^+^ and acquire CID breakdown curves for each complex (Figure 5). The primary unimolecular reaction of these complexes is simple dissociation of a hydrogen bond to form the protonated formate as seen previously for reactions involving methyl formate [23]. At higher collision energy the subsequent dissociation of the protonated formate ensues. In all cases dissociation of the initial complex occurs at very low collision energy, consistent with relatively weakly bound water molecules. The B3LYP/6-311+G(d,p) binding energies with respect to water loss for EF(W)H^+^, IF(W)H^+^, TF(W)H^+^, and PF(W)H^+^ are 1.01, 0.84, 0.82, and 0.99 eV, respectively (see Table S1 for all calculated dissociation energies).

EFH^+^ is known to create the CH_3_CH_2_OH_2_^+^ (*m*/*z* 47) ion when CO is lost [24]. Similarly, the loss of CO from PFH^+^, leads to the formation of C_6_H_7_O^+^ (*m*/*z* 95). This fragmentation is followed by a sequential loss of H_2_CO_2_ to form C_6_H_5_^+^ (*m*/*z* 77). In the case of IFH^+^, parallel loss of C_3_H_6_ and H_2_CO_2_ is seen, which results in the formation of H_3_CO_2_^+^ (*m*/*z* 47) and C_3_H_7_^+^ (*m*/*z* 43), respectively [24]. The loss of H_2_CO_2_ from TFH^+^ results in the C_4_H_9_^+^ (*m*/*z* 57) ion. The overall dominance of the loss of water from the formate–water complex is due to the much larger proton affinities (PA) of EF, IF, TF, and PF (calculated to be 799.4, 826.0, 841.1, and 793.1 kJ mol^−1^, respectively) compared to that of water (691 kJ mol^−1^) [26].

**Figure 6:**
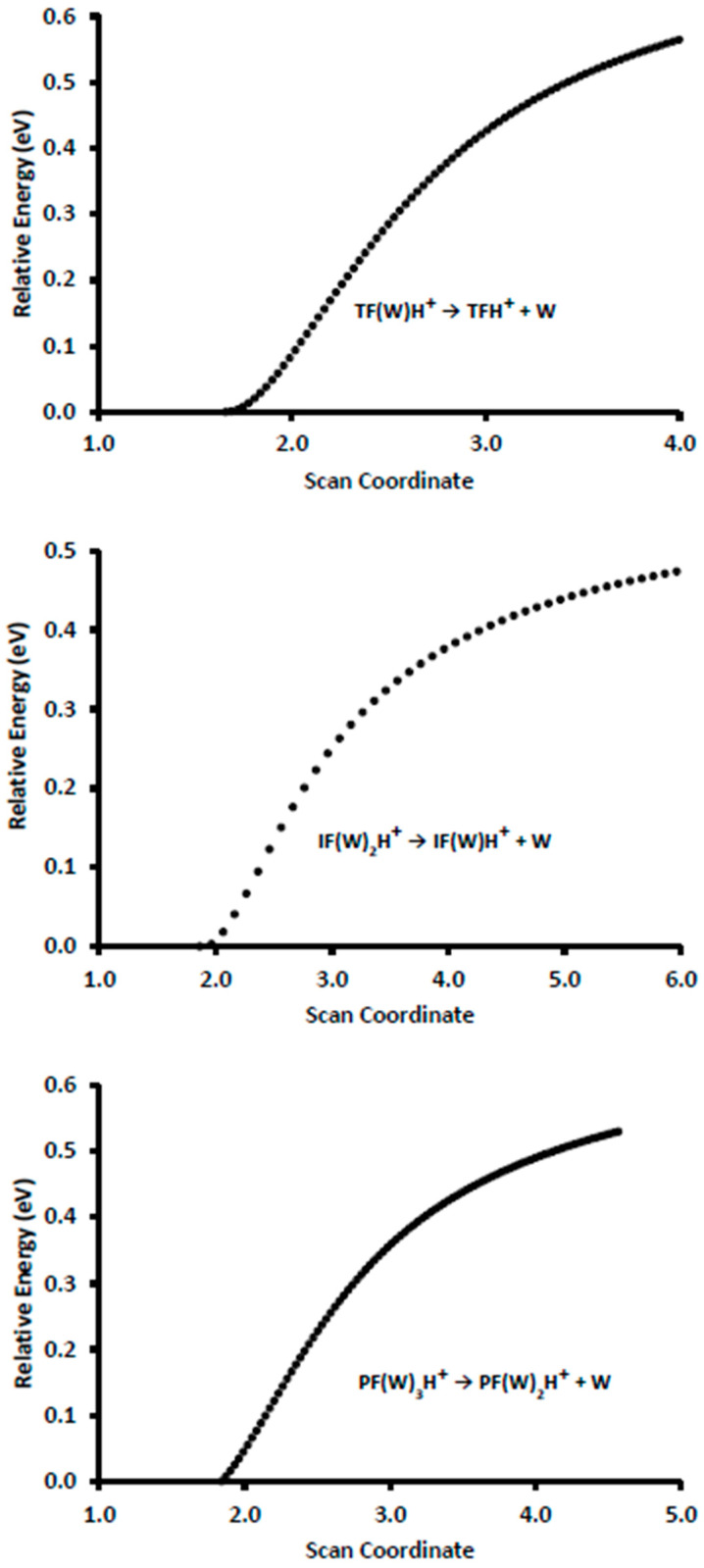
Relaxed potential energy scan of the dissociation of TF(W)H^+^, IF(W)_2_H^+^, and PF(W)_3_H^+^ at the B3LYP/6-311+G(d,p) level of theory.

Simple hydrogen bond dissociation is the main initial unimolecular process of these complexes. The possibility of reverse activation barriers resulting from the potential structural rearrangement of these complexes was investigated using relaxed potential energy scans of the dissociation for TF(W)H^+^, IF(W)_2_H^+^, and PF(W)_3_H^+^, shown as examples in Figure 6. All the scans from the proton-bound complexes of formates with one, two, and three water molecules clearly show that these complexes dissociate without a reverse barrier, which suggests that the relative product energies in Figure 3 control the relative abundance of the competing reactions.

**Figure 7:**
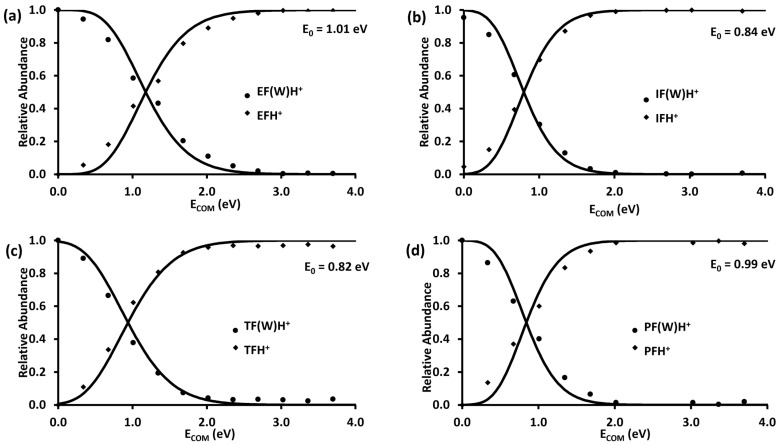
RRKM modeled breakdown curves for EF(W)_2_H^+^ IF(W)_2_H^+^, TF(W)_2_H^+^, or PF(W)_2_H^+^ complexes, employing the DFT calculated *E*_0_ values for the reactions and loose transition states with Δ^‡^S~20 J K^−1^ mol^−1^. Sequential fragmentation channels were summed into the primary channel to aid the modeling of those primary pathways. The solid line represents the RRKM fit. (**a**) EF(W)H^+^, (**b**) IF(W)H^+^, (**c**) TF(W)H^+^ and (**d**) PF(W)H^+^.

The initial dissociation pathways for the four proton-bound formate–water complexes were modeled with RRKM theory according to the semi-quantitative procedures outlined in the Experimental Procedures section. The results are summarized in Figure 7. For these experiments, the collision gas pressure was reduced so that no fragmentation occurred at 0 eV. The data in Figure 5 were obtained with higher gas pressure and one can see the effect of this near 0 eV collision energy where several of the precursor ions are already dissociating. This indicates that the gas is bleeding into the region between the first quadrupole and collision cell, prompting dissociation during the transmission between the two regions. The outcomes obtained using the model show that the calculated *E*_0_ values for the dissociation channels are consistent with the experimental data. In each case the entropy of activation needed to be significantly positive, consistent with a simple cleavage of the hydrogen bond.

## 3. Experimental Procedures

### 3.1. Chemicals

Water, ethyl formate, isopropyl formate, t-butyl formate, and phenyl formate were purchased from Sigma-Aldrich (Sigma-Aldrich, Oakville, CA, USA) and used without further purification.

### 3.2. Tandem Mass Spectrometry

All the studies reported herein were performed using a Micromass Quattro Ultima triple quadrupole mass spectrometer running the MassLynx (V 4.1) software system and outfitted with an electrospray ionization (ESI) source in a Z-spray configuration. For ion–molecule reactions, proton-bound water clusters (H_2_O)_2_H^+^ and (H_2_O)_3_H^+^ were generated in the electrospray source by delivering to the electrospray probe pure water (W6-1 Water, LOT 222746) with a syringe pump at a flow rate 50 μL/min. The capillary voltage was typically set to 3.5 kV but was adjusted to optimize ion yield. Nitrogen was used as the nebulizer gas with a flow rate of 100 L/h. The source and desolvation gas temperatures were held at 120 and 150 °C, respectively. The desired proton-bound water cluster ion was mass selected with the first quadrupole and transferred to the collision cell where it interacted with the chosen formate vapor introduced via a variable leak Granville-Phillips valve at room temperature [27]. The voltage at the entrance and exit of the collision cell was set at 50 V to give an extraction voltage for the derived reaction products, which were analyzed with the second quadrupole and continuous dynode electron multiplier detector. The resolution for both mass-selecting quadrupoles was set in the software to 17, yielding baseline resolution of neighboring masses.

Collision-induced dissociation (CID) experiments were carried out by first creating the desired ions in the electrospray source from a 1 μL/mL solution of formate in water. The ion of interest was mass selected with the first quadrupole and transmitted to the collision cell where it underwent collisions with argon target gas as a function of lab frame collision energy, E_LAB_ (generally between 0 and 30 eV, in 1 eV increments, for all the CID experiments). The argon target gas was kept at a pressure reading of 9.26 × 10^−6^ mbar. For these experiments, the entry and exit potentials of the collision cell were set to 0 V so that the lab frame collision energy better reflected the actual translational energy of the ions. Products of the CID process were determined by scanning the last quadrupole. Data were processed into breakdown curves by plotting the relative peak abundance in each mass spectrum as a function of center-of-mass collision energy, *E*_COM_. The latter was derived from the standard equation [28]:

(1) $$E_{COM}=E_{LAB}\left(\frac{m_{Ar}}{m_{Ar}+m_{i}}\right)$$

where *m*_Ar_ is the mass of argon and *m*_i_ is the mass of the colliding ion.

### 3.3. Computational Methods

The GAUSSIAN 16 (C.01) software package was used for all calculations [29]. The B3LYP hybrid density functional was employed along with the 6-311+G(d,p) basis set for all equilibrium geometry optimizations, harmonic vibrational frequency calculations, and transition state optimizations [30,31]. The intrinsic reaction coordinate approach in GAUSSIAN was used to confirm all the transition states. This level of theory was used previously with success when exploring the unimolecular chemistry of protonated formates [24] in which it compared favorably with the CBS-QB3 composite method. Structures, in the form of Gaussian archive entries for all calculated structures, are tabulated in the Supporting Information.

Kinetics calculations employed the standard Rice–Ramsperger–Kassel–Marcus (RRKM) equation for the microcanonical rate constant, *k*(*E*) [32,33].

(2) $$k\left(E\right)=\frac{\sigma N^{‡}(E-E_{0})}{h\rho (E)}$$

where σ represents the reaction degeneracy, h is Planck’s constant, $N^{‡}(E-E_{0})$ is the number of internal states for the transition state at internal energy $\left(E-E_{0}\right)$, and ρ(E) is the density of states for the reactant ion at internal energy (E) as calculated via the Beyer and Swinehart direct count algorithm [34]. For the qualitative comparisons in this study, vibrational sums and densities of states were employed in the RRKM calculations in the harmonic approximation. Once the RRKM *k*(*E*) rate curves were obtained, branching ratios were calculated as a function of center-of-mass collision energy. Our previous work modeling energy-resolved CID data employed a simple model in which the post-collision ions are assigned an effective temperature depending on the center-of-mass collision energy, and thus a “thermal” internal energy distribution, according to the relationship:

(3) *T*_eff_ = *T*_i_ + *α* × *E*_COM_

where *T*_i_ represents the initial temperature (300 K in the current study) and *α* describes the relationship between *E*_COM_ and the increase in the effective temperature (*T*_eff_)_._ This assumption limits the model to a purely semi-quantitative one for comparisons of related systems only [35].

## 4. Conclusions

Ion–molecule reactions between formates and proton-bound clusters of water demonstrate that the primary reaction product is the formation of the protonated formate due to its higher proton affinity. Loss of a water molecule from the encounter complex occurs without a reverse energy barrier in all cases. The binding energy of the encounter complexes is such that, when combined with their calculated internal energy distribution at 300 K, these encounter complexes would be stable under atmospheric conditions. In the low-pressure environment of the mass spectrometer, however, the rate constants for the dissociation of the encounter complexes are too great for them to be observed. The results support the conclusion that when formates interact with atmospheric water, these encounter complexes can go on to lose a water molecule, but more energy would be required to form the protonated formate.

## Data Availability

Data is available from the corresponding author.

## Supplementary Materials

The following supporting information can be downloaded at: https://www.mdpi.com/article/10.3390/molecules28114431/s1, Figure S1. Calculated minimum energy structures for (W)_2_H^+^ and (W)_3_H^+^ at the B3LYP/6-311+G(d,p) level of theory; Figure S2. Vibrational internal energy distributions at 300 K for the encounter complexes; Figure S3. RRKM *k*(*E*) vs. E curves for the loss of water from the encounter complexes; Table S1. Calculated dissociation energies for all reactions in Figure 3; Gaussian archive entries for all structures reported in this study.
