# Supplementary material for: On the Gas-Phase Interactions of Alkyl and Phenyl Formates with Water: Ion–Molecule Reactions with Proton-Bound Water Clusters
Source: Molecules. 2023 May 30;28(11):4431. doi: 10.3390/molecules28114431 (PMC10254361; doi:10.3390/molecules28114431)
Supplement: Supplementary file 1 [file molecules-28-04431-s001.zip › molecules-2409878-supplementary.pdf]

On the Gas-Phase Interactions of Alkyl and Phenyl Formates with Water: Ion–Molecule Reactions with Proton-Bound Water Clusters

Malick Diedhiou and Paul M. Mayer *

Department of Chemistry and Biomolecular Sciences, University of Ottawa, Ottawa, ON K1N 6N5, Canada; mdiedhio@uottawa.ca
* Correspondence: pmmayer@uottawa.ca

Supporting Information

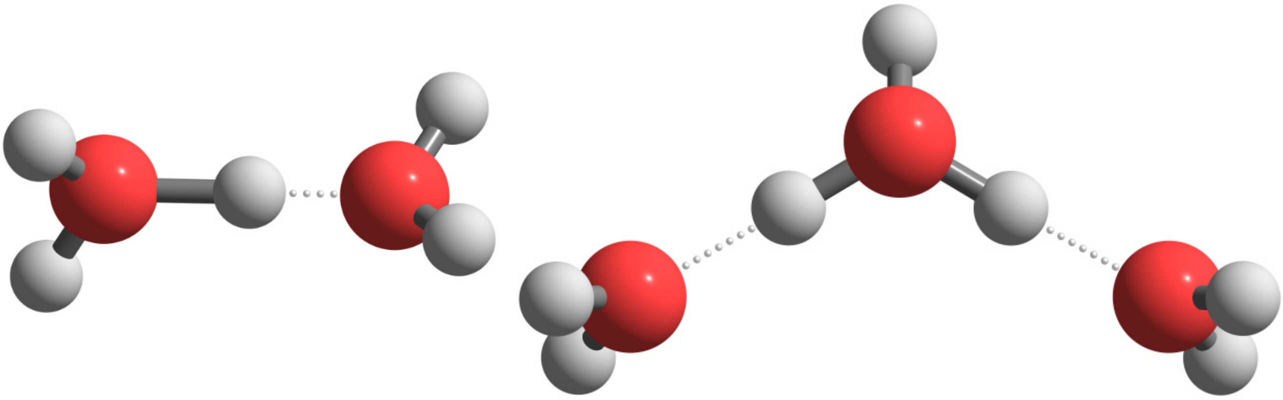

Figure S1. Calculated minimum energy structures for $(W)_2H^+$ and $(W)_3H^+$ at the B3LYP/6-311+G(d,p) level of theory.

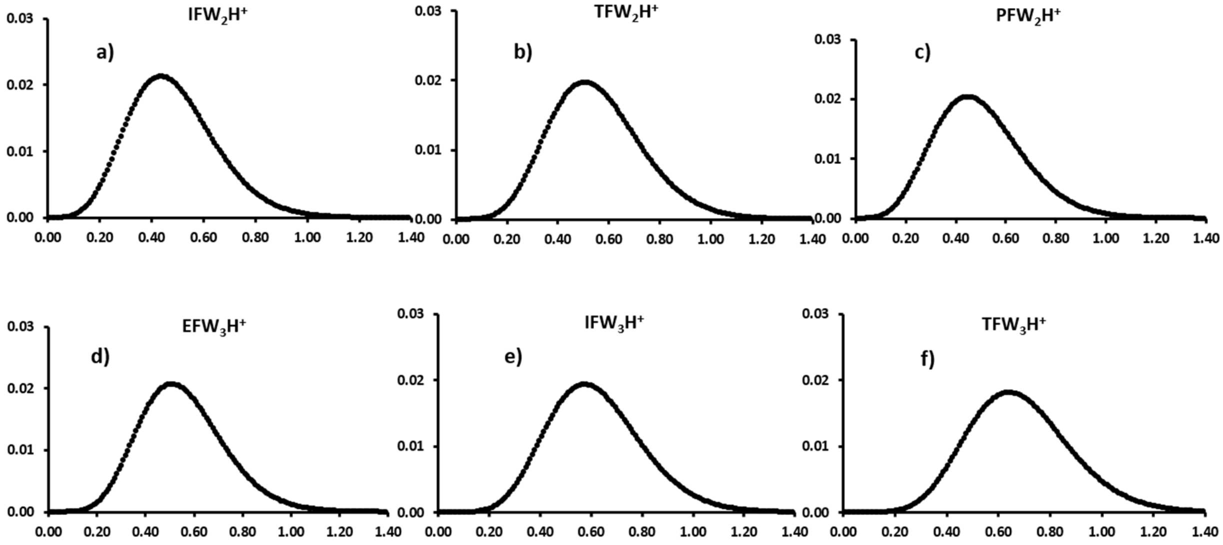

Figure S2. Vibrational internal energy distributions at 300 K for the encounter complexes.

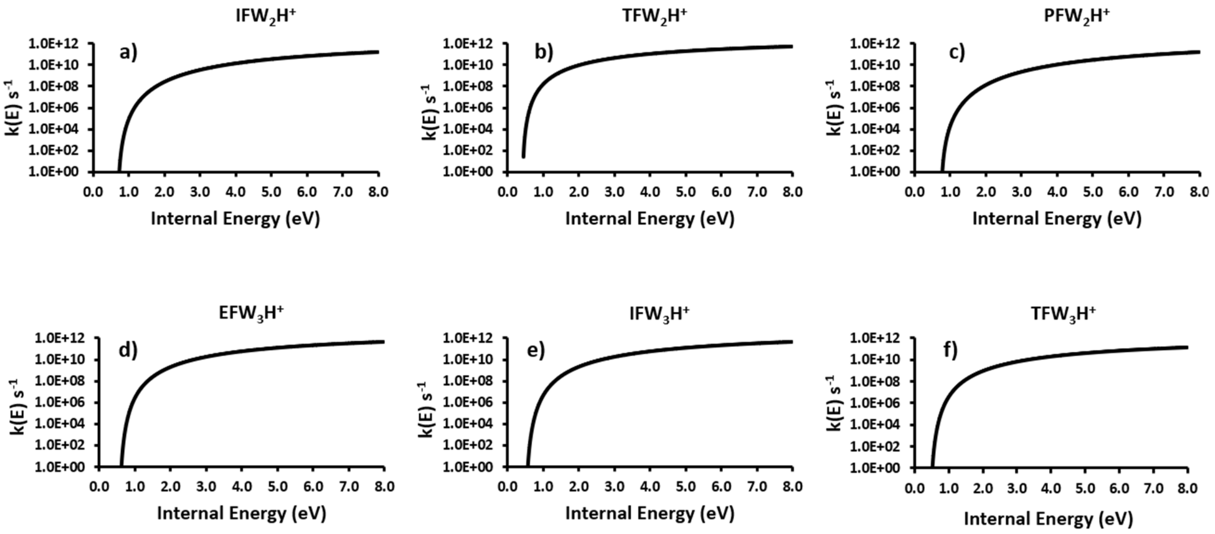

Figure S3. RRKM k(E) vs E curves for the dissociation of the ion-molecule encounter complexes

Table S1. Theoretical dissociation energies calculated for the reactions in Figure 3.

Reaction	Theoretical E_0 (eV)
$EF(W)_2H^+ \rightarrow EF(W)H^+ + W$	0.74
$\rightarrow EFH^+ + 2W$	1.01
$\rightarrow WH^+ + W + EF$	2.22
$EF(W)_3H^+ \rightarrow EF(W)_2H^+ + W$	0.59
$\rightarrow EF(W)H^+ + 2W$	0.74
$\rightarrow (W)_2H^+ + W + EF$	1.40
$\rightarrow EFH^+ + 3W$	1.02
$IF(W)_2H^+ \rightarrow IF(W)H^+ + W$	0.72
$\rightarrow IFH^+ + 2W$	0.84
$\rightarrow WH^+ + W + IF$	2.32
$IF(W)_3H^+ \rightarrow IF(W)_2H^+ + W$	0.56
$\rightarrow IF(W)H^+ + 2W$	0.75
$\rightarrow (W)_2H^+ + W + IF$	1.48
$\rightarrow IFH^+ + 3W$	0.84
$TF(W)_2H^+ \rightarrow TF(W)H^+ + W$	0.46
$\rightarrow TFH^+ + 2W$	0.82
$\rightarrow WH^+ + W + TF$	2.46
$TF(W)_3H^+ \rightarrow TF(W)_2H^+ + W$	0.51
$\rightarrow TF(W)H^+ + 2W$	0.45
$\rightarrow (W)_2H^+ + W + TF$	1.35
$\rightarrow TFH^+ + 3W$	0.83
$PF(W)_2H^+ \rightarrow PF(W)H^+ + W$	0.75
$\rightarrow PFH^+ + 2W$	0.99
$\rightarrow WH^+ + W + PF$	2.13
$PF(W)_3H^+ \rightarrow PF(W)_2H^+ + W$	0.61
$\rightarrow PF(W)H^+ + 2W$	0.75
$\rightarrow (W)_2H^+ + W + PF$	1.32
$\rightarrow PFH^+ + 3W$	0.99

W

1\1\GINC-GRA1223\FOpt\RB3LYP\6-311+G(d,p)\H2O1\MALICK10\09-Feb-2022\0\
\# opt freq b3lyp/6-311+g(d,p) nosymm\\water_neut\\0,1\O,-1.6260818951
,0.7586864469,-1.4164208634\H,-0.8096692423,1.2094120869,-1.6522714104
\H,-2.2081575426,1.4508200561,-1.0886300262\\Version=ES64L-G16RevC.01\
HF=-76.4584639\RMSD=9.770e-09\RMSF=7.527e-05\Dipole=0.1702318,0.830219
5,0.0667893\Quadrupole=-0.4923083,2.0991452,-1.6068369,-2.4122926,-1.4
864117,-1.8794545\PG=CS [SG(H2O1)]\\@

(W)H$^+$

1\1\GINC-GRA387\FOpt\RB3LYP\6-311+G(d,p)\H3O1(1+)\MALICK10\19-Apr-2022
\0\\# opt freq b3lyp/6-311+g(d,p) nosymm\\Pro_water\\1,1\O,-1.40507673
08,-0.714879444,0.1982995364\H,-1.5087228604,-1.6308944763,-0.13441781
89\H,-0.7032507135,-0.6176275311,0.8754541607\H,-1.3589426953,-0.04201
75485,-0.5131328782\\Version=ES64L-G16RevC.01\HF=-76.7310717\RMSD=1.20
1e-09\RMSF=1.948e-04\Dipole=0.4780939,-0.1085975,-0.2720813\Quadrupole
=0.8369278,0.9998033,-1.8367311,3.6099743,1.2542589,-0.3615289\PG=C01
[X(H3O1)]\\@

(W)$_2$H$^+$

1\1\GINC-GRA1029\FOpt\RB3LYP\6-311+G(d,p)\H5O2(1+)\MALICK10\09-Feb-202
2\0\\# opt freq b3lyp/6-311+g(d,p) nosymm\\Pro_water_di\\1,1\O,-1.5722
942624,0.9414300439,-1.4826754551\H,-0.8252147121,1.2966531793,-1.9862
373061\H,-2.2616575086,1.6125258194,-1.3678584838\O,-1.3470844464,-0.7
496601306,0.1944702389\H,-1.5005713111,-1.6718618084,-0.0600042822\H,-
0.7170550246,-0.6942457956,0.92786727\H,-1.4112832247,0.110349352,-0.6
361302718\\Version=ES64L-G16RevC.01\HF=-153.2480721\RMSD=2.408e-09\RMS
F=1.367e-05\Dipole=0.4262866,0.1275319,0.0716095\Quadrupole=-0.2183589
,1.5262596,-1.3079007,-1.9868039,3.6085192,-5.4707103\PG=C01 [X(H5O2)]
\\@

(W)$_3$H$^+$

1\1\GINC-GRA1029\FOpt\RB3LYP\6-311+G(d,p)\H7O3(1+)\MALICK10\09-Feb-202
2\0\\# opt freq b3lyp/6-311+g(d,p) nosymm\\Pro_water_tri\\1,1\O,-1.317
8122106,1.2038541764,-1.3721176301\H,-0.5442226056,1.6191850555,-1.928
6909667\H,-1.9941860884,1.8427913726,-1.1075355625\O,-0.7811504034,-0.
5008808408,0.362281468\H,-1.0626357013,-1.420593316,0.280941873\H,-0.3
308752674,-0.3932492175,1.2087110305\H,-1.0721093812,0.5201728702,-0.6
274972729\O,0.498640323,2.1583158582,-2.7869934619\H,1.1936932245,2.78
4645653,-2.5517201637\H,0.5711613304,1.9618105684,-3.7292295936\\Versi
on=ES64L-G16RevC.01\HF=-229.7460364\RMSD=7.361e-09\RMSF=3.303e-05\Dipo
le=0.1531081,0.2401541,0.2715141\Quadrupole=-6.5180181,1.8817343,4.636
2837,1.2698697,-2.3279846,-12.6279852\PG=C01 [X(H7O3)]\\@

EF

1\1\GINC-GRA111\FOpt\RB3LYP\6-311+G(d,p)\C3H6O2\MALICK10\27-Aug-2020\0
\\# opt freq b3lyp/6-311+g(d,p) nosymm\\EF_neut\\0,1\C,-0.6593736545,1
.3003938586,0.4088038738\H,-0.2027546928,0.4551529128,0.9274198874\H,-
0.7243671754,1.0601272658,-0.6541826634\H,-0.010713745,2.1721527582,0.
528331867\C,-2.0313171592,1.6003145003,0.9759839237\H,-2.497054847,2.4
432814857,0.4556944008\H,-1.973455904,1.8359368302,2.0433824869\O,-2.8
517205682,0.4195408389,0.7948403391\C,-4.1198501658,0.4819413012,1.237
4685666\H,-4.3784271948,1.4548573695,1.6955011585\O,-4.8876659833,-0.4
30027081,1.1418899195\\Version=ES64L-G16RevC.01\HF=-268.4604029\RMSD=4
.358e-09\RMSF=3.582e-05\Dipole=1.045466,1.4404532,0.2063049\Quadrupole
=-10.4104104,5.7002277,4.7101827,-9.1293584,0.639485,2.8476322\PG=C01
[X(C3H6O2)]\\@

(EF)H$^+$

1\1\GINC-GRA701\FOpt\RB3LYP\6-311+G(d,p)\C3H7O2(1+)\MALICK10\26-Mar-20
21\0\\# opt freq b3lyp/6-311+g(d,p) nosymm\\Prot_EF\\1,1\C,-1.35632000
99,0.0378661429,0.8862382693\H,-0.7985462023,0.3446679855,1.7688838581
\H,-1.2223947756,0.7578575601,0.0770822943\O,-2.7996801193,0.161508185

8,1.3500585607\C,-3.5898207397,0.9817059314,0.8382334643\H,-3.28203521
09,1.6502583652,0.0292025344\O,-4.7861586544,1.014999503,1.2974491295\
H,-5.3549492765,1.6705851848,0.8587493584\C,-1.1131107019,-1.391598676
9,0.4854390656\H,-1.7094583967,-1.6807666256,-0.3814216775\H,-0.056910
4336,-1.4843432582,0.2164190494\H,-1.3121571492,-2.0741140879,1.312423
3034\\Version=ES64L-G16RevC.01\HF=-268.7782425\RMSD=5.851e-09\RMSF=2.7
00e-05\Dipole=-0.4159163,1.3789772,-0.8608516\Quadrupole=25.4587596,-9
.2015946,-16.2571649,-16.4506882,-3.7627209,1.7416002\PG=C01 [X(C3H7O2
)]\\@
EF(W)H$^+$
1\1\GINC-GRA600\FOpt\RB3LYP\6-311+G(d,p)\C3H9O3(1+)\MALICK10\22-Jan-20
23\0\\# opt freq b3lyp/6-311+g(d,p) nosymm\\EF+Water\\1,1\C,-1.3785295
996,-0.2997005421,-0.2242406815\H,-1.4871742143,-0.9535331674,-1.08771
79877\H,-1.7366967535,0.7031155244,-0.4660364374\O,-2.327734089,-0.887
7320984,0.7788131473\C,-3.3317631018,-0.2388185059,1.1859159268\H,-3.5
246372957,0.7719970278,0.8121933459\O,-4.1031067647,-0.7763895459,2.02
68641676\H,-4.9031788515,-0.1890188971,2.3152919525\H,-6.0995174997,1.
0611858449,3.592321017\O,-6.0432485617,0.6814820988,2.7057803633\H,-6.
9446225417,0.4745881504,2.4263962634\C,0.0103985257,-0.3183758458,0.36
12157852\H,0.3099343111,-1.3320736404,0.6302102727\H,0.7046993332,0.05
11625025,-0.3981295862\H,0.0902119432,0.3274674042,1.237455721\\Versio
n=ES64L-G16RevC.01\HF=-345.2758926\RMSD=4.359e-09\RMSF=1.948e-05\Dipol
e=-1.3547931,1.192356,0.1219742\Quadrupole=43.8807806,-29.6541987,-14.
2265818,-8.1251399,-29.2102524,5.2878185\PG=C01 [X(C3H9O3)]\\@
EF(W)$_2$H$^+$
1\1\GINC-GRA703\FOpt\RB3LYP\6-311+G(d,p)\C3H11O4(1+)\MALICK10\23-Jan-2
023\0\\# opt freq b3lyp/6-311+g(d,p) nosymm\\EF+Water_dimer\\1,1\C,-0.
7963342225,0.4637786031,-0.0163754815\H,-0.1203143352,0.3293093858,0.8
264983042\H,-0.8854154393,1.5288940402,-0.2412433223\O,-2.1092162378,-
0.0081739512,0.4898354916\C,-3.1309793284,0.7851147346,0.4725212288\H,
-2.986573709,1.8016422797,0.0909763731\O,-4.2434040932,0.4351624054,0.
872174953\H,-4.5309720165,-0.7593789086,1.2959741403\H,-5.8909446853,-
1.9085628051,1.6389594109\O,-4.8852277989,-1.7697780919,1.6331738327\H
,-4.4929102406,-2.076559518,2.4610836555\O,-7.4077545818,-2.1925846883
,1.6353659323\H,-7.8295666539,-2.8887814585,1.1183273038\H,-8.10428416
66,-1.6315611255,1.9954438842\C,-0.3973228736,-0.3574700119,-1.2198573
043\H,0.595825783,-0.0394373954,-1.5471313038\H,-0.3489574854,-1.41918
41792,-0.9729237154\H,-1.090175805,-0.2151590451,-2.0516981729\\Versio
n=ES64L-G16RevC.01\HF=-421.7627494\RMSD=8.507e-09\RMSF=3.368e-06\Dipol
e=-1.2177089,-0.4603606,0.6927737\Quadrupole=56.6599309,-27.2694518,-2
9.3904791,25.3824051,-23.6470044,-7.5849306\PG=C01 [X(C3H11O4)]\\@
EF(W)$_3$H$^+$
1\1\GINC-GRA744\FOpt\RB3LYP\6-311+G(d,p)\C3H13O5(1+)\MALICK10\22-Jan-2
023\0\\# opt freq b3lyp/6-311+g(d,p) nosymm\\EF+Water_trimer\\1,1\C,-1
.7171673123,-2.6118472346,0.533694819\H,-0.668398679,-2.3170106138,0.5
547170916\H,-2.1583705256,-2.2942586571,-0.4149424222\O,-2.3472905988,
-1.848691365,1.6219909931\C,-3.2827866,-0.9874845911,1.3518703437\H,-3
.5558069705,-0.8847200138,0.29086994\O,-3.8203243249,-0.3354244881,2.2
367157137\H,-4.8701406772,0.6906229022,2.2487217381\H,-5.2300010305,2.
2930812853,2.687043875\O,-5.5983225111,1.4196958875,2.3482493397\H,-6.
3912381763,1.1072365996,2.8837846365\O,-4.6676205192,3.6933511877,3.16
0388984\H,-4.7930425962,4.5277678927,2.6943986774\H,-4.1042887799,3.86
81737777,3.9225928615\O,-7.6757592171,0.6231059485,3.6694410237\H,-8.5
791504459,0.6664293707,3.3360891459\H,-7.7052979468,0.198907773,4.5343
240801\C,-1.9006826104,-4.0913535406,0.7883494466\H,-1.3779691534,-4.6
541579845,0.0108998069\H,-1.4813769463,-4.3744554378,1.7550652962\H,-2
.9552984588,-4.3735945486,0.7638660798\\Version=ES64L-G16RevC.01\HF=-4
98.2474434\RMSD=9.701e-09\RMSF=4.900e-06\Dipole=-1.5373002,1.77393,0.2

953759\Quadrupole=52.3584617,-24.6232659,-27.7351959,-19.1517327,-51.8
907199,17.2346598\PG=C01 [X(C3H13O5)]\\@

IF

1\1\GINC-GRA1085\FOpt\RB3LYP\6-311+G(d,p)\C4H8O2\MALICK10\18-Apr-2021\
0\\# opt freq rb3lyp/6-311+g(d,p) nosymm\\IF_neut\\0,1\C,-0.7754980253
,1.207028206,0.3716112893\H,-0.4124066121,0.2784610312,0.8186821479\H,
-0.8860201683,1.0502540015,-0.7036609353\H,-0.0244044822,1.9853317938,
0.5307959046\C,-2.099382184,1.6168650567,1.0019701931\O,-3.0618562144,
0.550899726,0.7644214006\C,-4.1359636896,0.8044863306,-0.0016297537\C,
-2.0159073676,1.841957235,2.5050804898\H,-2.9890285131,2.124733726,2.9
122044195\H,-1.6841268452,0.9308110041,3.0087095602\H,-1.3031645,2.640
3228395,2.7281316149\O,-4.9686283699,-0.0172767025,-0.2521968572\H,-4.
1703137338,1.8459156462,-0.3727318761\H,-2.4750792946,2.5243801156,0.5
138193925\\Version=ES64L-G16RevC.01\HF=-307.7902483\RMSD=9.130e-09\RMS
F=5.604e-07\Dipole=1.2266635,1.3365165,0.3160144\Quadrupole=-12.604140
9,7.5479978,5.056143,-7.9923124,-1.5754173,1.2719843\PG=C01 [X(C4H8O2)
]\\@

(IF)H$^+$

1\1\GINC-GRA1120\FOpt\RB3LYP\6-311+G(d,p)\C4H9O2(1+)\MALICK10\20-Sep-2
020\0\\# opt freq b3lyp/6-311+g(d,p) nosymm\\IF_ion_1\\1,1\C,-0.759346
6112,1.1971030609,0.3637565092\H,-0.418061794,0.2683814462,0.824326139
9\H,-0.8828349961,1.0493307614,-0.7103088464\H,0.0177790317,1.95277792
65,0.5087383274\C,-2.0208752878,1.7010882349,1.0171970964\O,-3.0992284
083,0.5840598627,0.7500930617\C,-4.1359249161,0.8155526521,0.091303945
7\C,-1.9848096278,1.8663919334,2.5151102154\H,-2.94956168,2.1785803969
,2.9182564176\H,-1.6620974806,0.9475700686,3.007802072\H,-1.2575484252
,2.6492208293,2.7481017476\O,-5.0179853678,-0.0872910047,-0.1299064508
\H,-4.7921244357,-0.9522685333,0.2649706125\H,-4.3637108428,1.79157558
7,-0.338921655\H,-2.4293504683,2.5789145579,0.5113376668\\Version=ES64
L-G16RevC.01\HF=-308.1170758\RMSD=8.920e-09\RMSF=6.929e-06\Dipole=-0.5
379135,-0.0079182,-0.3029305\Quadrupole=23.0975942,-8.6605006,-14.4370
936,-7.0176267,-4.8644132,3.2868496\PG=C01 [X(C4H9O2)]\\@

IF(W)H$^+$

1\1\GINC-GRA286\FOpt\RB3LYP\6-311+G(d,p)\C4H11O3(1+)\MALICK10\22-Jan-2
023\0\\# opt freq b3lyp/6-311+g(d,p) nosymm\\IF+Water\\1,1\C,-1.406271
4317,-0.2798636392,-0.2467249909\H,-1.9830776468,0.5335866106,-0.69587
04259\O,-2.1818912359,-0.6713068618,1.0150309963\C,-3.2471836269,-0.08
08031726,1.3339500506\H,-3.6411593276,0.7284122554,0.7104607685\O,-3.8
554322002,-0.4359946706,2.384895983\H,-4.715501469,0.0940584602,2.5751
876078\H,-6.0228523229,1.5259376086,3.5454039692\O,-5.9673286595,0.893
3391522,2.8176210962\H,-6.8411623475,0.4929012421,2.7227607879\C,-0.05
13117172,0.1931706647,0.229688427\H,0.481373906,-0.6065690321,0.747363
0641\H,0.5380990143,0.4889102978,-0.6416599427\H,-0.1313302555,1.05763
74912,0.8912298398\C,-1.3975252298,-1.5099782798,-1.1261290188\H,-0.85
57458224,-1.2744097824,-2.045423295\H,-0.8881694101,-2.339297156,-0.63
20501063\H,-2.4069796975,-1.8214130881,-1.4006111405\\Version=ES64L-G1
6RevC.01\HF=-384.6092965\RMSD=3.384e-09\RMSF=1.015e-05\Dipole=-1.75448
72,1.1644106,0.2836782\Quadrupole=40.895896,-26.8209818,-14.0749142,-8
.9279437,-29.1021195,7.5575218\PG=C01 [X(C4H11O3)]\\@

IF(W)$_2$H$^+$

1\1\GINC-GRA399\FOpt\RB3LYP\6-311+G(d,p)\C4H13O4(1+)\MALICK10\22-Jan-2
023\0\\# opt freq b3lyp/6-311+g(d,p) nosymm\\IF+Water_dimer\\1,1\C,-0.
9666757777,0.6176153113,-0.363246834\H,-0.9696504761,1.6956983142,-0.1
795737131\O,-2.2266226275,0.0885464695,0.2891850817\C,-3.0430223273,0.
8938254919,0.8622651051\H,-2.8070527647,1.9617924567,0.8847648835\O,-4
.0955446061,0.509914368,1.4009153789\H,-4.4184636165,-0.6293951845,1.3
691540996\H,-5.8497064153,-1.882197692,1.4205617902\O,-4.8572883887,-1
.7691442571,1.3382853505\H,-4.4105771023,-2.4236916817,1.8885485687\O,

-7.4352899452,-2.1053967573,1.5107838847\H,-7.969428997,-2.5420972282,
0.8376748407\H,-8.0375094453,-1.7813731801,2.1898604702\C,0.1952691435
,-0.0516575375,0.3402066781\H,1.1290653742,0.314480905,-0.0928487027\H
,0.2065568126,0.1773305676,1.4075130101\H,0.160201104,-1.1348670435,0.
2071660458\C,-1.0859375346,0.3154325656,-1.8423864247\H,-1.9585324229,
0.800408233,-2.2841191743\H,-0.1963495625,0.6918522465,-2.3528880202\H
,-1.1499481945,-0.7605048472,-2.0166801886\\Version=ES64L-G16RevC.01\H
F=-461.0946641\RMSD=5.652e-09\RMSF=5.823e-06\Dipole=-1.2641386,-0.4694
574,0.5627908\Quadrupole=51.6969856,-24.0028115,-27.694174,22.4936552,
-19.8117391,-6.5525212\PG=C01 [X(C4H13O4)]\\@
IF(W)$_3$H$^+$
1\1\GINC-GRA600\FOpt\RB3LYP\6-311+G(d,p)\C4H15O5(1+)\MALICK10\23-Jan-2
023\0\\# opt freq b3lyp/6-311+g(d,p) nosymm\\IF+Water_trimer\\1,1\C,-2
.1965157161,-2.8890258468,0.3823763612\H,-2.5298046517,-2.3833806827,-
0.5298355308\O,-2.7993524036,-2.1483104423,1.5259562074\C,-3.548166759
1,-1.1133857133,1.3065005842\H,-3.6992550091,-0.8351558228,0.252672349
3\O,-4.0561293209,-0.4853758477,2.2294070447\H,-4.9107876585,0.6814855
487,2.2475976989\H,-5.0774673985,2.2742189913,2.834651546\O,-5.5259760
149,1.5176310966,2.3480807901\H,-6.4239317609,1.2955169936,2.741197754
3\O,-4.3708574754,3.5049270455,3.5487899809\H,-4.3044986343,4.39520601
37,3.1854588239\H,-3.8940940707,3.4974613182,4.3862944063\O,-7.8716543
248,0.9594487189,3.3032136082\H,-8.7025396684,1.1984019803,2.877139062
1\H,-8.0871628058,0.4621145579,4.1000671089\C,-2.7387444655,-4.3050702
492,0.4364183202\H,-2.3211933244,-4.8846177825,-0.390114831\H,-2.45690
53128,-4.7902497316,1.3732021729\H,-3.8268428375,-4.3201157891,0.34673
97395\C,-0.6901209929,-2.7846950937,0.5310165871\H,-0.2070827201,-3.31
65737956,-0.2918837569\H,-0.3569166057,-1.7451645127,0.5061461808\H,-0
.3636214885,-3.2357272848,1.470372592\\Version=ES64L-G16RevC.01\HF=-53
7.5785464\RMSD=6.418e-09\RMSF=9.426e-06\Dipole=-1.7387586,2.319215,0.5
662498\Quadrupole=53.3493032,-25.2415326,-28.1077706,-18.9913952,-48.1
101585,20.6392863\PG=C01 [X(C4H15O5)]\\@
TF
1\1\GINC-GRA139\FOpt\RB3LYP\6-311+G(d,p)\C5H10O2\MALICK10\29-Aug-2020\
0\\# opt freq b3lyp/6-311+g(d,p) nosymm\\TBF_neut\\0,1\C,-0.7073088796
,1.2358042246,0.4009888925\H,-0.2986980269,0.3706849699,0.9268128993\H
,-0.8183419844,0.9752258343,-0.6533654847\H,0.0018266942,2.063055079,0
.4842645126\C,-2.0559120114,1.6346007399,0.9970649516\O,-2.8748798278,
0.4202929446,0.8019999652\C,-4.1519520762,0.3851666817,1.2087213415\H,
-4.5086572031,1.3139889115,1.6815823461\O,-4.846517501,-0.5821278337,1
.067007741\C,-1.9213959166,1.9353343787,2.4929623211\H,-2.8710603957,2
.2338476046,2.9427731689\H,-1.5464069471,1.0585330177,3.0255527959\H,-
1.2141522502,2.7551507135,2.6423186646\C,-2.6685943728,2.8044416005,0.
2209561781\H,-1.9969878632,3.6657985566,0.2610143131\H,-2.8137489614,2
.5320871175,-0.8268048009\H,-3.6302661267,3.117431119,0.6338763642\\Ve
rsion=ES64L-G16RevC.01\HF=-347.1140208\RMSD=4.062e-09\RMSF=2.327e-05\D
ipole=1.0246877,1.6240393,0.2842954\Quadrupole=-10.5080558,5.7003431,4
.8077127,-10.38971,0.1367552,3.2152101\PG=C01 [X(C5H10O2)]\\@
(TF)H$^+$
1\1\GINC-GRA95\FOpt\RB3LYP\6-311+G(d,p)\C5H11O2(1+)\MALICK10\29-Aug-20
20\0\\# opt freq b3lyp/6-311+g(d,p) nosymm\\TBF_ion_1\\1,1\C,-0.739175
646,1.1665653036,0.3847297486\H,-0.4099532127,0.2868712328,0.938161501
9\H,-0.9342339781,0.8954821025,-0.6530509198\H,0.0756599611,1.89715993
17,0.3956816498\C,-1.9369383367,1.8160909141,1.0277737585\O,-3.0363789
038,0.5094594431,0.8903854179\C,-4.214268155,0.444726787,1.2525235016\
C,-1.8078613982,2.0562610112,2.5111780148\H,-2.7160933146,2.4630213043
,2.954200329\H,-1.4983830261,1.1549802494,3.0422485428\H,-1.0152912739
,2.8002268038,2.643428433\C,-2.5607774644,2.9305636856,0.2255006033\H,
-1.825549052,3.741085253,0.1849559456\H,-2.7640925999,2.625588857,-0.8

020143385\H,-3.46318553,3.3297128385,0.6869432971\O,-4.8283400815,1.43
33642816,1.8316457584\H,-5.7504958259,1.2242490983,2.0546317242\H,-4.7
551093623,-0.4888961676,1.0737083218\\Version=ES64L-G16RevC.01\HF=-347
.4457267\RMSD=7.134e-09\RMSF=9.214e-06\Dipole=-1.4306753,-0.454168,0.2
964904\Quadrupole=33.1137197,-15.9564791,-17.1572406,-10.4230974,-18.9
490915,5.0876126\PG=C01 [X(C5H11O2)]\\@

TF(W)H$^+$

1\1\GINC-GRA644\FOpt\RB3LYP\6-311+G(d,p)\C5H13O3(1+)\MALICK10\22-Jan-2
023\0\\# opt freq b3lyp/6-311+g(d,p) nosymm\\TF+Water\\1,1\C,-1.308220
8419,0.0127029309,-0.22974466\O,-1.9466794542,0.0941927805,1.219323563
7\C,-3.1860369525,0.1444776472,1.4088185382\H,-3.8895532856,0.13477040
43,0.5722791372\O,-3.6239509869,0.2079334035,2.5989384606\H,-4.6433162
471,0.2458145617,2.6632838422\H,-6.6316381511,1.1046160527,2.978460544
8\O,-6.168486168,0.3006382917,2.7107651139\H,-6.6850904455,-0.44516598
79,3.0415966772\C,0.1700724044,-0.0200366891,0.1056212352\H,0.41778219
62,-0.8916278126,0.7128438631\H,0.7386632018,-0.0780423602,-0.82540102
73\H,0.4744051746,0.8826762064,0.6368964882\C,-1.8108141466,-1.2766246
997,-0.856131712\H,-1.3027445522,-1.4120969572,-1.8139007823\H,-1.5781
924022,-2.1400699928,-0.2305056263\H,-2.8834528953,-1.2555817278,-1.06
48455056\C,-1.7295969108,1.2735205424,-0.9646271388\H,-2.8016830959,1.
3025638215,-1.1751187227\H,-1.4418823017,2.1704507126,-0.4134327241\H,
-1.2154254094,1.2952726617,-1.9284747352\\Version=ES64L-G16RevC.01\HF=
-423.9379351\RMSD=5.119e-09\RMSF=8.924e-06\Dipole=-2.0997219,0.0802998
,0.1908864\Quadrupole=42.816336,-25.7246816,-17.0916545,-3.2325214,-28
.5160886,1.2698772\PG=C01 [X(C5H13O3)]\\@

TF(W)$_2$H$^+$

1\1\GINC-GRA396\FOpt\RB3LYP\6-311+G(d,p)\C5H15O4(1+)\MALICK10\22-Jan-2
023\0\\# opt freq b3lyp/6-311+g(d,p) nosymm\\TF+Water_dimer\\1,1\C,-0.
9697544169,0.7230945747,-0.3136751836\O,-2.3527769528,0.2793234078,0.2
709301606\C,-3.411838289,0.9528632217,0.0630315418\H,-3.3785453479,1.8
747631228,-0.5187750346\O,-4.529169174,0.6046635706,0.5103758318\H,-4.
5983970702,-0.3476055632,1.0755642092\H,-5.7229094279,-1.8664650801,1.
7435809329\O,-4.8014896595,-1.4963996459,1.6996274771\H,-4.3548026431,
-1.6730803355,2.5348615251\O,-7.2361503043,-2.5252859554,1.803377654\H
,-7.4895054209,-3.3676657645,1.4100108642\H,-8.0339715789,-2.137559775
3,2.1794676143\C,-1.098526351,0.7097319903,-1.8292007112\H,-1.77997688
51,1.4787771172,-2.2008414474\H,-0.116842779,0.915299058,-2.2621573461
\H,-1.4259192784,-0.266873083,-2.1903939125\C,-0.6485692151,2.09124629
44,0.2683435375\H,-1.3219952114,2.8726669378,-0.091681142\H,-0.6681189
214,2.0730097296,1.3596208113\H,0.3605285391,2.3709388979,-0.042989184
9\C,-0.0541718152,-0.3694151452,0.2092352304\H,-0.3628235235,-1.349804
0583,-0.1567220387\H,0.9622621763,-0.1780635018,-0.1415840497\H,-0.040
6645298,-0.3835772647,1.3002102505\\Version=ES64L-G16RevC.01\HF=-500.4
224888\RMSD=2.394e-09\RMSF=4.391e-06\Dipole=-1.1138667,-0.5103813,0.52
14448\Quadrupole=44.3461276,-20.12706,-24.2190675,21.2271974,-19.62521
18,-8.0341718\PG=C01 [X(C5H15O4)]\\@

TF(W)$_3$H$^+$

1\1\GINC-GRA738\FOpt\RB3LYP\6-311+G(d,p)\C5H17O5(1+)\MALICK10\23-Jan-2
023\0\\# opt freq b3lyp/6-311+g(d,p) nosymm\\TF+Water_trimer\\1,1\C,-2
.044548143,-2.7289996207,0.2645721145\O,-2.5059747793,-1.8355355872,1.
4075362331\C,-3.5275956633,-1.0540626711,1.2847092999\H,-4.054710718,-
1.0564414366,0.3225881949\O,-3.8906074249,-0.3363326846,2.216163261\H,
-4.9710566817,0.5779706794,2.2878657895\H,-5.4204802089,2.2090454316,2
.5663904163\O,-5.7523767639,1.27374017,2.4201828179\H,-6.4053440215,0.
9951043788,3.1289687042\O,-4.9122073565,3.7151632903,2.7412971192\H,-5
.2102716758,4.4685627105,2.2193726531\H,-4.2456867877,4.0369769548,3.3
584326097\O,-7.4878922,0.5403087085,4.2144992975\H,-8.439000427,0.4903
77157,4.0670013398\H,-7.3187005396,0.2363493704,5.1131976148\C,-3.1703

9695,-3.7022232675,-0.0715825421\H,-2.8092057531,-4.423938493,-0.80747
07105\H,-3.4856392629,-4.2548884269,0.8157178516\H,-4.0395621347,-3.20
38525102,-0.5082359619\C,-0.8442978893,-3.4376935785,0.8769065943\H,-0
.4119356913,-4.1221250593,0.1440145077\H,-0.0781777038,-2.7189547913,1
.1723089351\H,-1.1400609862,-4.013866121,1.7551681763\C,-1.6439668424,
-1.8412113432,-0.9097566432\H,-2.4973782306,-1.3227330864,-1.354022143
1\H,-0.8992137261,-1.103536518,-0.6043336364\H,-1.2018354085,-2.462796
8059,-1.6914332333\\Version=ES64L-G16RevC.01\HF=-576.9044879\RMSD=4.42
3e-09\RMSF=1.200e-06\Dipole=-2.1640539,2.1881747,0.9154518\Quadrupole=
49.860358,-23.7926765,-26.0676814,-17.0825759,-52.6785669,15.6774488\P
G=C01 [X(C5H17O5)]\\@
PF
1\1\GINC-GRA1198\FOpt\RB3LYP\6-311+G(d,p)\C7H6O2\MALICK10\02-Sep-2020\
0\\# opt freq b3lyp/6-311+g(d,p) nosymm\\PF_neut\\0,1\O,-2.5477326759,
0.2881141242,0.3017080688\C,-3.6056371103,-0.2744569445,0.9495412839\O
,-3.9999957309,-1.3683051806,0.6876323482\H,-4.0421630226,0.3914254326
,1.7116875814\C,-2.0825534552,1.5273362124,0.7434958284\C,-2.013021749
5,2.5605573505,-0.1853049344\C,-1.6485209834,1.7017645438,2.0543391071
\C,-1.5080930221,3.7956438762,0.2126690234\H,-2.3486355067,2.384567464
7,-1.199927268\C,-1.1543306715,2.9468413052,2.4434024672\H,-1.67517781
05,0.8730045474,2.7521904347\C,-1.0827267524,3.9933013811,1.5267556253
\H,-1.4489831583,4.6052667818,-0.5054626772\H,-0.8146208411,3.09067578
13,3.4625012344\H,-0.6918019994,4.9567145339,1.8319108268\\Version=ES6
4L-G16RevC.01\HF=-420.9168961\RMSD=8.987e-09\RMSF=3.134e-05\Dipole=0.4
220365,1.5535169,0.5766711\Quadrupole=-6.9237206,2.3936203,4.5301003,-
7.5116046,-1.0951861,1.3610738\PG=C01 [X(C7H6O2)]\\@
(PF)H$^+$
1\1\GINC-GRA107\FOpt\RB3LYP\6-311+G(d,p)\C7H7O2(1+)\MALICK10\02-Sep-20
20\0\\# opt freq b3lyp/6-311+g(d,p) nosymm\\PF_ion_1\\1,1\O,-2.7206704
336,0.3416995293,0.3740005319\C,-3.6305844977,-0.2060597947,1.04445930
55\O,-4.0255125482,-1.3597999662,0.6530614301\H,-4.749162163,-1.725203
1527,1.1901580801\H,-4.0609428984,0.2837962345,1.9208154325\C,-2.14957
7961,1.6099994195,0.7977213113\C,-2.0827569443,2.5925188994,-0.1718958
63\C,-1.6632680471,1.7354975662,2.0878397421\C,-1.5166798618,3.8098432
785,0.2024626574\H,-2.4572175291,2.4173508337,-1.1723559023\C,-1.10089
07392,2.9644295873,2.4349773549\H,-1.6725660126,0.908909059,2.78921205
17\C,-1.0328716583,3.9945143801,1.4985244658\H,-1.4511890313,4.6098645
263,-0.5242755498\H,-0.7016949848,3.1026112856,3.431975417\H,-0.587754
3195,4.9420927143,1.7755249847\\Version=ES64L-G16RevC.01\HF=-421.23123
97\RMSD=6.187e-09\RMSF=3.195e-05\Dipole=-1.8353266,-1.5316027,0.814585
8\Quadrupole=19.5704539,-5.7883093,-13.7821445,3.8388534,-15.2580572,0
.9029021\PG=C01 [X(C7H7O2)]\\@
PF(W)H$^+$
1\1\GINC-GRA720\FOpt\RB3LYP\6-311+G(d,p)\C7H9O3(1+)\MALICK10\22-Jan-20
23\0\\# opt freq b3lyp/6-311+g(d,p) nosymm\\PF+Water\\1,1\O,-2.1675784
688,-0.688164858,0.972442853\C,-3.2289126565,-0.062475836,1.283115825\
H,-3.6274142112,0.6988433964,0.606895563\O,-3.7927629366,-0.3457582716
,2.3730839744\H,-4.6627254317,0.1832053839,2.5600048902\H,-5.94911291,
1.6107267447,3.5141410781\O,-5.9018786238,0.9574256655,2.8040668218\H,
-6.7720181482,0.5430591703,2.7377406572\C,-1.4558344782,-0.3344267209,
-0.2337803226\C,-1.2249568186,-1.3595254594,-1.1329947316\C,-0.9947638
579,0.9615895247,-0.3934442658\C,-0.5090961613,-1.0475398841,-2.287403
8403\H,-1.5877031975,-2.3607362132,-0.9379757759\C,-0.2818668201,1.249
7333375,-1.5580196883\H,-1.1454075582,1.7163965073,0.3697958963\C,-0.0
439863024,0.2507655704,-2.4998070071\H,-0.3130075629,-1.8235838355,-3.
0167766141\H,0.0980366755,2.2516360223,-1.7145939985\H,0.5163990285,0.
4811323857,-3.3974478646\\Version=ES64L-G16RevC.01\HF=-497.7287653\RMS
D=5.199e-09\RMSF=8.015e-06\Dipole=-2.6900997,0.9816285,1.4165879\Quadr

upole=37.1542834,-25.4909881,-11.6632952,-8.2788058,-32.87277,8.179187
4\PG=C01 [X(C7H9O3)]\\@

PF(W)$_2$H$^+$

1\1\GINC-GRA402\FOpt\RB3LYP\6-311+G(d,p)\C7H11O4(1+)\MALICK10\23-Jan-2
023\0\\# opt freq b3lyp/6-311+g(d,p) nosymm\\PF+Water_dimer\\1,1\O,-2.
2897186659,0.1344242238,0.0662246147\C,-2.9977831161,0.8135083531,0.92
7530641\H,-2.6406261443,1.8123113073,1.1957828142\O,-4.0240751711,0.35
29107253,1.4212575853\H,-4.5508034192,-0.8252944684,1.102755416\H,-5.9
97565419,-1.8786363944,1.3093488903\O,-5.0869944509,-1.7613860446,0.86
76322772\H,-4.5500177886,-2.5603106173,0.9528970752\O,-7.3873814397,-2
.0987517482,1.9059003915\H,-8.1900585698,-2.1905893558,1.3790800894\H,
-7.6513440546,-1.9578544134,2.8225427113\C,-1.0697811872,0.7028093579,
-0.4263907902\C,-0.0201148223,0.9256535649,0.4509308922\C,-0.990468001
1,0.9476897805,-1.7870649316\C,1.1656306582,1.4461634493,-0.068661078\
H,-0.1089628195,0.6834926742,1.5036419725\C,0.2039638303,1.4629935784,
-2.2876447177\H,-1.8346084964,0.7409470426,-2.4329516971\C,1.276202483
6,1.7140166641,-1.4315151807\H,2.0019009031,1.6306160776,0.5943790948\
H,0.293350089,1.6670163669,-3.3475588959\H,2.2012815616,2.113280846,-1
.8288076841\\Version=ES64L-G16RevC.01\HF=-574.2162261\RMSD=4.457e-09\R
MSF=3.960e-06\Dipole=-3.0847902,-1.619103,1.4069123\Quadrupole=57.8879
368,-29.6753066,-28.2126302,29.2289185,-25.3413438,-9.0883037\PG=C01 [
X(C7H11O4)]\\@

PF(W)$_3$H$^+$

1\1\GINC-GRA402\FOpt\RB3LYP\6-311+G(d,p)\C7H13O5(1+)\MALICK10\22-Jan-2
023\0\\# opt freq b3lyp/6-311+g(d,p) nosymm\\PF+Water_trimer\\1,1\O,-2
.865212844,-2.1846597547,1.4252525273\C,-3.5596757968,-1.0971872982,1.
1917293876\H,-3.6573538507,-0.8136381834,0.1350102771\O,-4.055958085,-
0.4655184253,2.1103591404\H,-4.8922382945,0.7573444739,2.2017994689\H,
-5.0085545114,2.2758073003,2.9475462733\O,-5.4668704441,1.5976861393,2
.3596012649\H,-6.3978100879,1.380179773,2.6773072059\O,-4.2948786895,3
.3782617157,3.8185110501\H,-4.1565313304,4.2932921515,3.5487425671\H,-
3.8458388208,3.2525002043,4.6620173884\O,-7.8812012578,1.0638973823,3.
1088752228\H,-8.669092002,1.3647236146,2.64202854\H,-8.1722096141,0.53
06008932,3.8570628458\C,-2.2487676771,-2.8494164533,0.3294954234\C,-2.
6345518339,-4.1576607771,0.0818835574\C,-1.2579450012,-2.2066322315,-0
.398377897\C,-2.0098436577,-4.8421290629,-0.9587214303\H,-3.3971735664
,-4.6247770917,0.6924128095\C,-0.6469075203,-2.9049856245,-1.440285934
6\H,-0.9483091802,-1.1991082866,-0.1459015347\C,-1.0222374136,-4.21710
82415,-1.7201872915\H,-2.2951034494,-5.8653138059,-1.1709204003\H,0.13
04950042,-2.4237068477,-2.0210863123\H,-0.5392644651,-4.7564683837,-2.
5257940293\\Version=ES64L-G16RevC.01\HF=-650.7012528\RMSD=3.409e-09\RM
SF=4.371e-06\Dipole=-2.6215935,3.4339442,1.7518542\Quadrupole=52.53182
12,-25.3781404,-27.1536808,-19.0724541,-50.2054796,26.3300111\PG=C01 [
X(C7H13O5)]\\@
